# Supplementary figures and images for: Small molecule inhibitors and a kinase-dead expressing mouse model demonstrate that the kinase activity of Chk1 is essential for mouse embryos and cancer cells
Source: Life Sci Alliance. 2020 Jun 22;3(8):e202000671. doi: 10.26508/lsa.202000671 (PMC7335382; doi:10.26508/lsa.202000671)

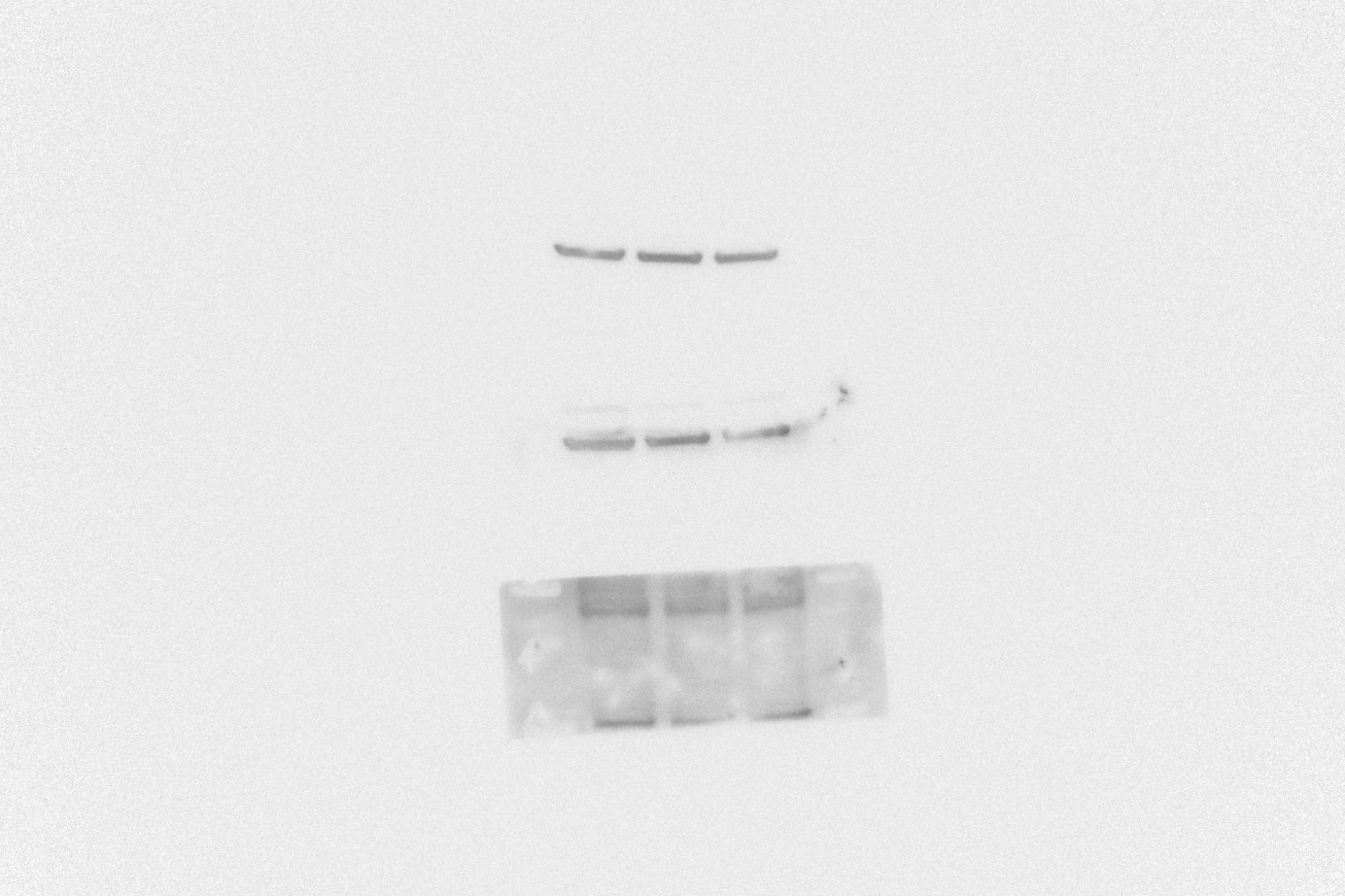

Supplement: Supplementary file 1 [file LSA-2020-00671_SdataF1.zip › Uncropped blots/Fig 1b ACTIN.tif]

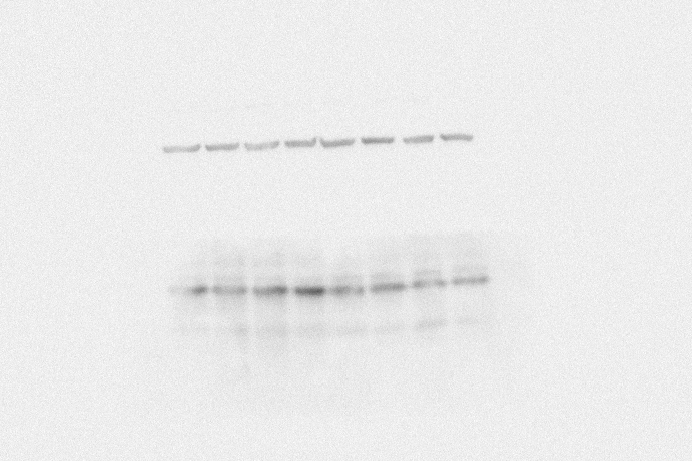

Supplement: Supplementary file 1 [file LSA-2020-00671_SdataF1.zip › Uncropped blots/Fig 1c actin.tif]

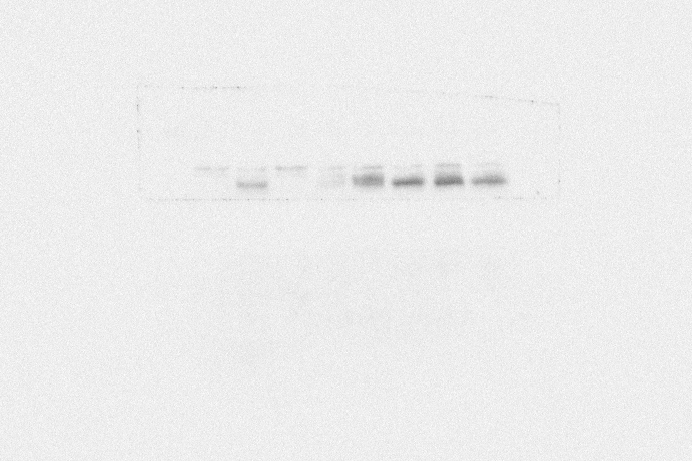

Supplement: Supplementary file 1 [file LSA-2020-00671_SdataF1.zip › Uncropped blots/Fig 1c RPA2.tif]

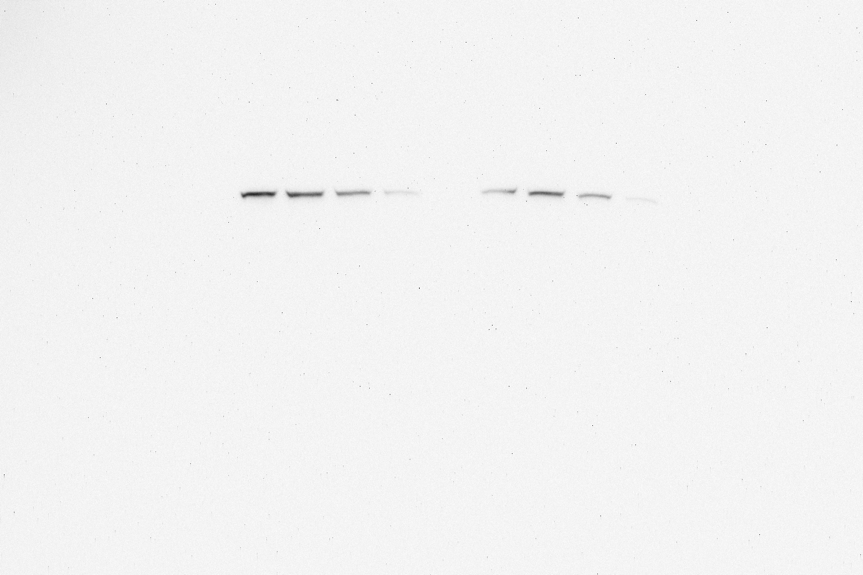

Supplement: Supplementary file 1 [file LSA-2020-00671_SdataF1.zip › Uncropped blots/Fig 1A chk1.png]

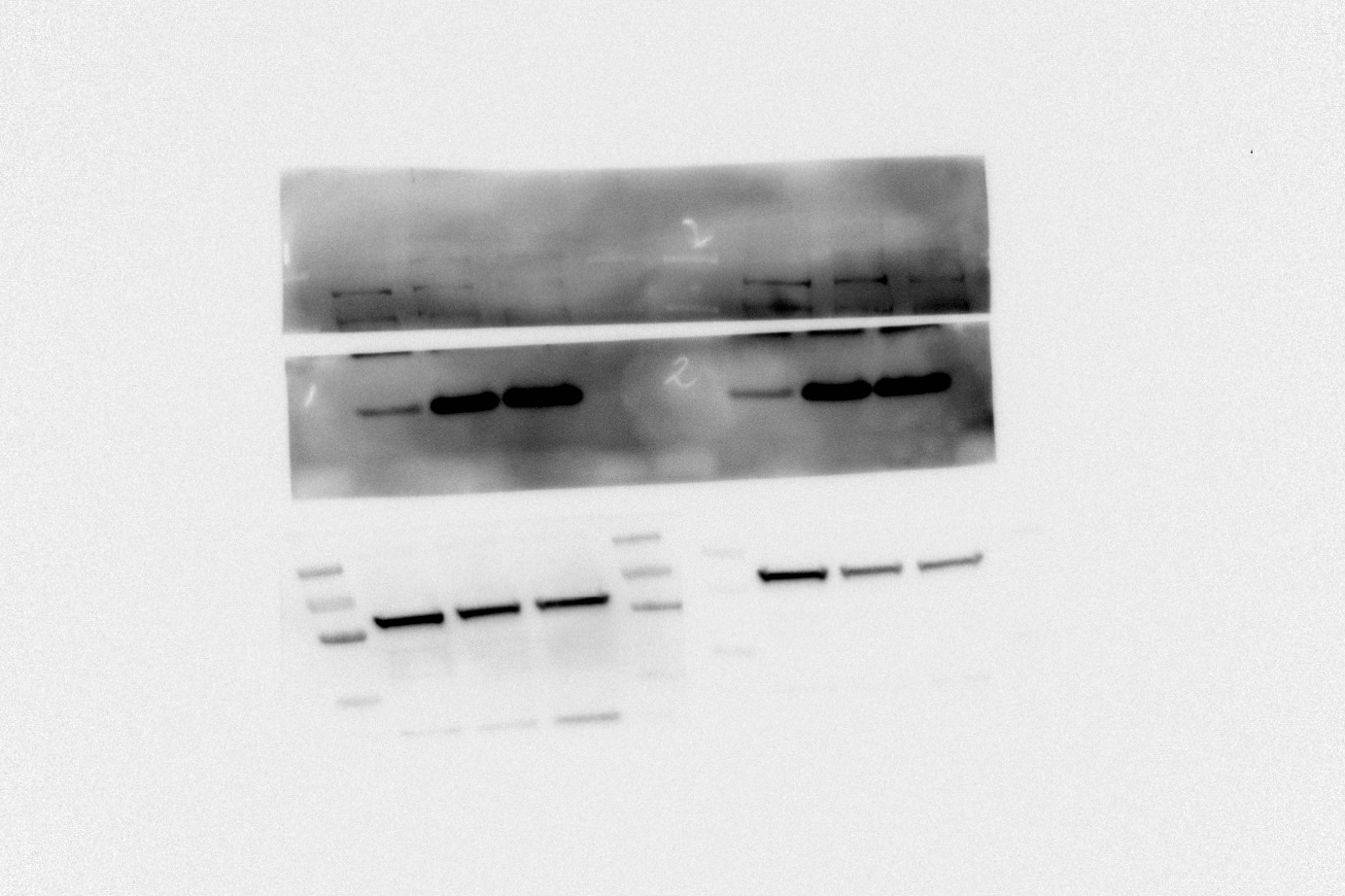

Supplement: Supplementary file 1 [file LSA-2020-00671_SdataF1.zip › Uncropped blots/Fig 1b Chk1 1.tif]

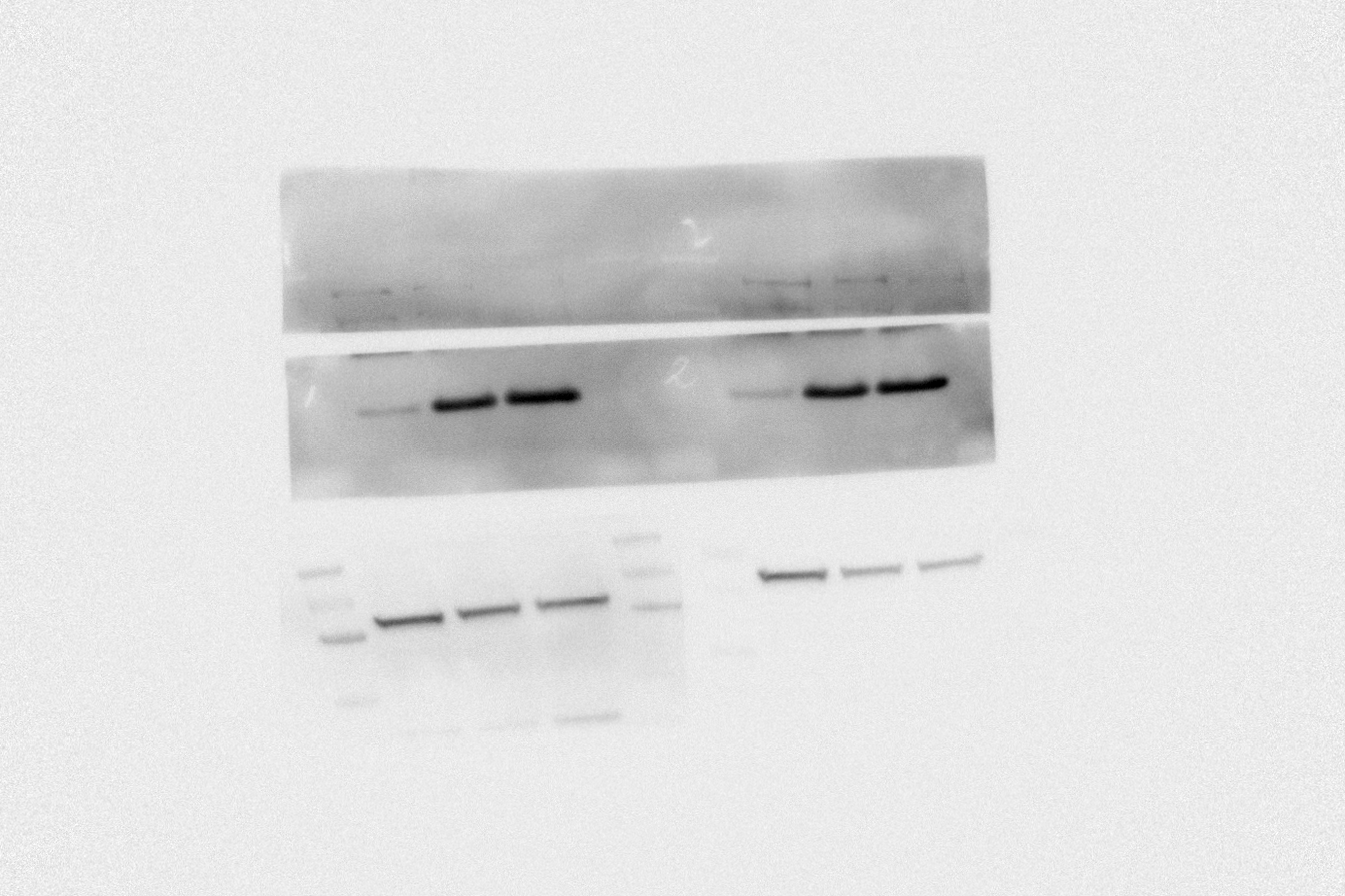

Supplement: Supplementary file 1 [file LSA-2020-00671_SdataF1.zip › Uncropped blots/Fig 1B pH2AX .tif]

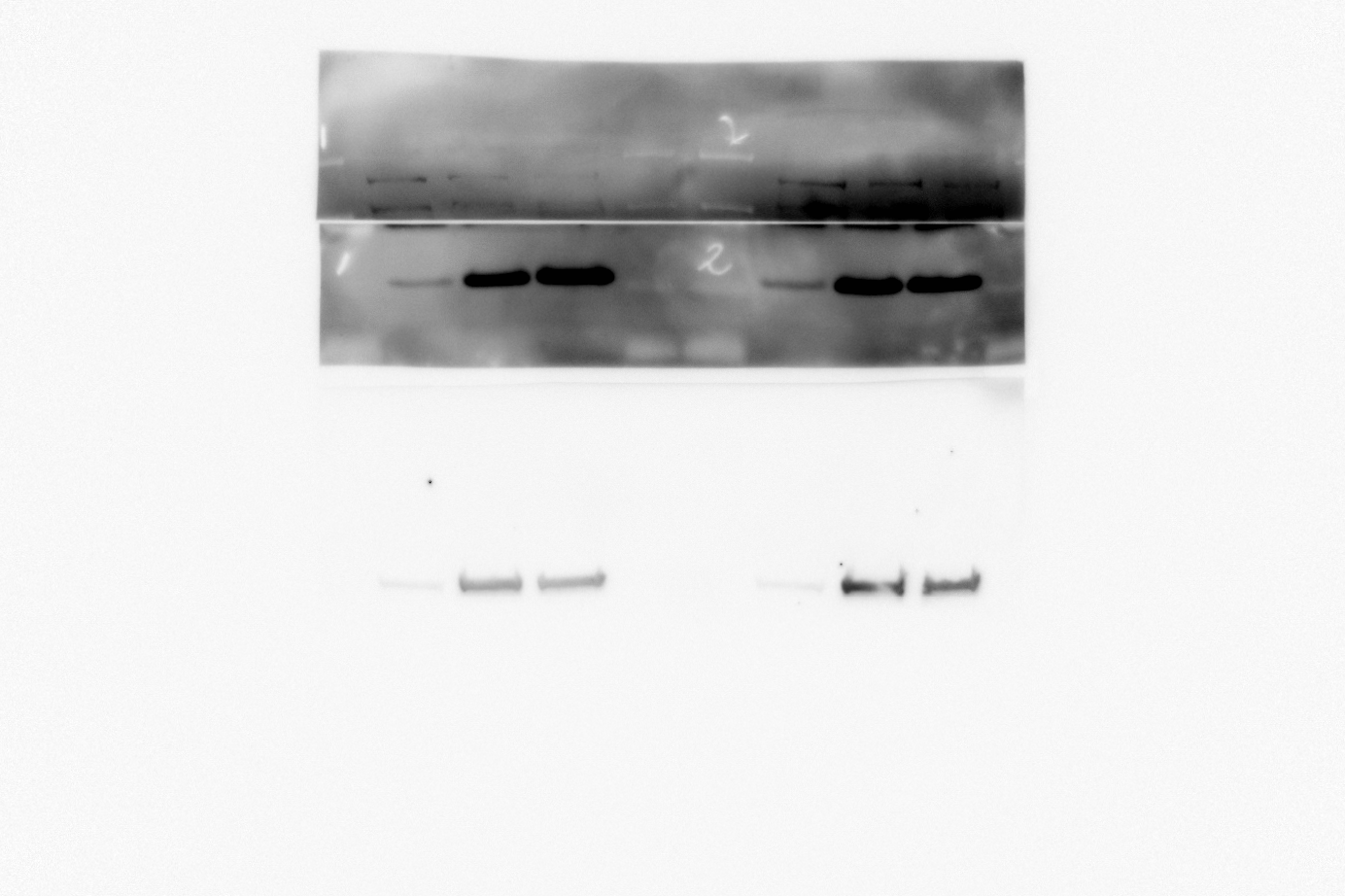

Supplement: Supplementary file 1 [file LSA-2020-00671_SdataF1.zip › Uncropped blots/Fig 1b cPARP 1.tif]

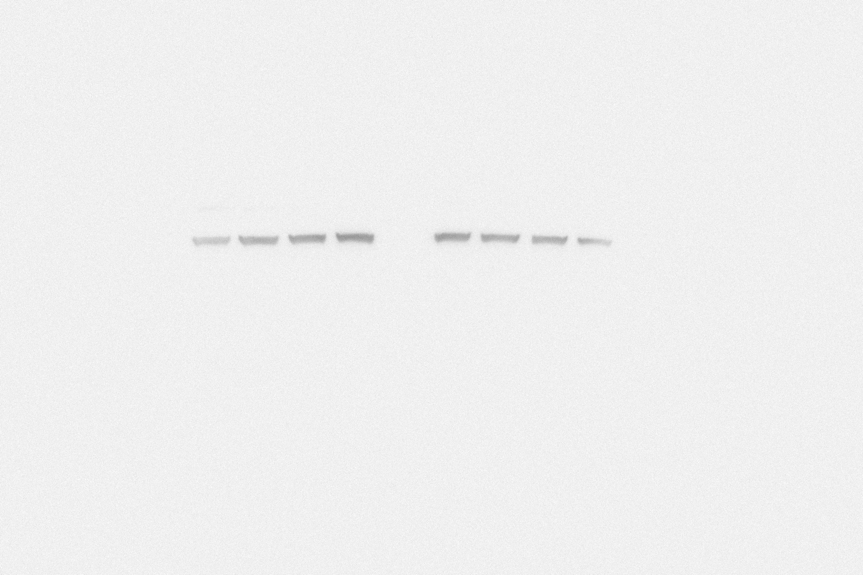

Supplement: Supplementary file 1 [file LSA-2020-00671_SdataF1.zip › Uncropped blots/Fig 1A actin.png]

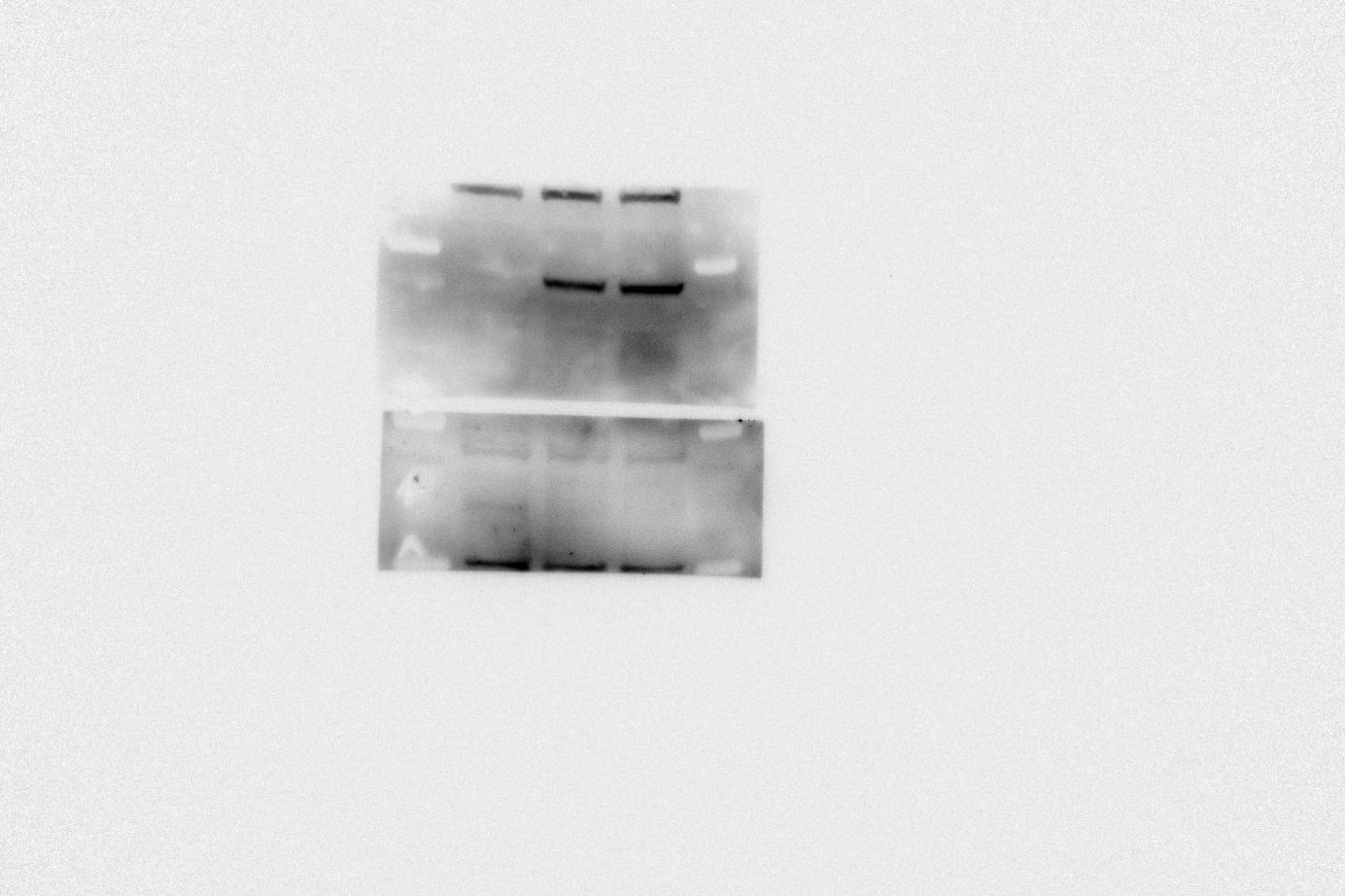

Supplement: Supplementary file 1 [file LSA-2020-00671_SdataF1.zip › Uncropped blots/Fig 1B pChk1.tif]

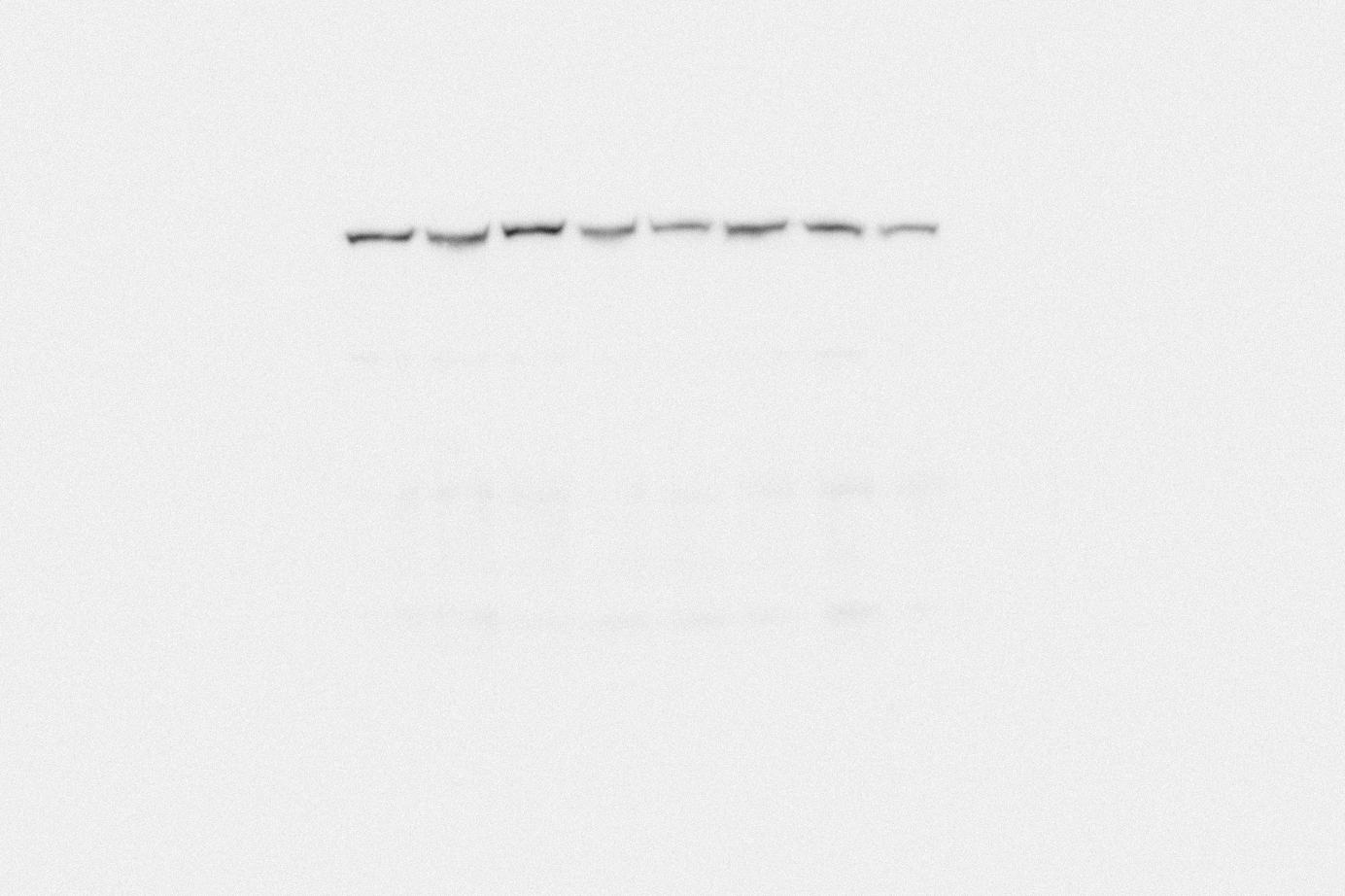

Supplement: Supplementary file 1 [file LSA-2020-00671_SdataF1.zip › Uncropped blots/Fig 1c Chk1.tif]

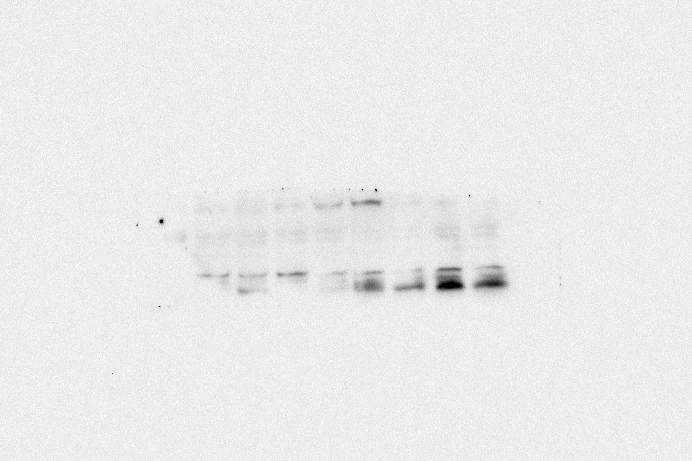

Supplement: Supplementary file 1 [file LSA-2020-00671_SdataF1.zip › Uncropped blots/Fig 1c pCHK1.tif]
